# Quartz Crystal Microbalance Aptasensor for Sensitive Detection of Mercury(II) Based on Signal Amplification with Gold Nanoparticles

**DOI:** 10.3390/s120607080

**Published:** 2012-05-29

**Authors:** Zong-Mu Dong, Guang-Chao Zhao

**Affiliations:** 1 College of Environmental Science and engineering, Anhui Normal University, Wuhu 241000, China; E-Mail: dzongmu@mail.ahnu.edu.cn; 2 Anhui Key Laboratory of Chem-Biosensing, School of Chemistry and Materials Science, Anhui Normal University, Wuhu 241000, China

**Keywords:** mercury ion, aptamer, gold nanoparticle, quartz crystal microbalance, flow system

## Abstract

We show that a short mercury-specific aptamer (MSA) along with gold nanoparticles (Au-NPs) can be used to determine Hg(II) ion by a combination of a QCM-based sensor and a flow system. The MSA binds specifically to Hg(II), and the Au-NPs can amplify the signal to enhance sensitivity. Specifically, the short thiolated MSAs are immobilized on the surface of the QCM as the capture probe, and the MSAs are linked to the Au-NPs as the linking probe. The two components can form a sandwich structure of the T-Hg(II)-T type in the presence of Hg(II) ions. This leads to change in the mass on the QCM and a change in the resonance frequency. Hg(II) can be determined with a detection limit of 0.24 ± 0.06 nM which is better by three orders of magnitude than previous methods. The sensor can be regenerated by disrupting the T-Hg(II)-T base pairs with a solution of cysteine.

## 1. Introduction

Mercury is a highly toxic heavy metal ion that exists in metallic, inorganic, and organic forms [1]. Solvated mercuric ion (Hg^2+^), one of the inorganic forms of mercury, mostly exists in surface water due to its high water solubility and high stability, which can be ingested by aquatic life and eventually enters human beings through the food chain [2]. As a toxic ion, Hg^2+^ can cause the disruption of cell membranes [3], the impairment of mitochondrial function [4], and the inhibition of DNA replication in a cell [5]. Therefore, it is critical to determine and quantify Hg^2+^ under aqueous conditions with high sensitivity and selectivity.

Indeed, there are numerous reports on Hg^2+^ detection by using Hg^2+^-sensitive fluorophores or chromophores [6–8]. Recently, the aptamer-based Hg^2+^ detection assays, which use the coordinate interaction between thymine (T) and Hg^2+^, have attracted significant interest. Aptamers are nucleic acid-based (DNA or RNA) affinity probes, which can provide the conjugate interaction between Hg^2+^ and thymine [9–11]. Based on this, several novel Hg^2+^ detection assays in aqueous media have been developed [12–17]. For example, a fluorescence resonance energy transfer (FRET) sensor for Hg^2+^ was designed by using a mercury-specific aptamer (MSA) probe labeled with fluorophore/quencher units [16]. MSA modified gold nanoparticles (Au-NPs) were also employed as a colorimetric probe [17], optical probe [18], surface enhanced raman spectroscopy (SERS) [19], surface plasmon resonance (SPR) spectra probe [20] and electrochemical probe for Hg^2+^ [12,15]. In addition, conjugating polymers [21] and DNAzymes [22–24] were also exploited to couple with MSA for Hg^2+^ detection. Because of the specific interaction between Hg^2+^ and thymine, these assays have a satisfying selectivity. However, most of these approaches rely on labels or multiple washing steps [25]. Furthermore, fluorescence-based aptasensors suffer from stability and photobleaching of fluorophores. On the other hand, electrochemical-based aptasensor must be labeled with redox species that are employed as signal transducers [26,27]. Label-free monitoring of biorecognition events provides a promising platform, which is simple, cost-effective, and requires no external modification on the biomolecules. The QCM-based sensor is one of the promising candidates for the development of label-free sensors.

A QCM is an acoustic sensor based on a piezoelectric crystal. The characteristics of QCM-based sensors have been described by Sauerbrey [28]. The sensor is sensitive and allows for noninvasive on-line measurements of adsorption and biophysical changes. However, it is well known that the signal response of QCM-based assays is unstable when the target concentration in a sample is very low, which results in low sensitivity and dissatisfactory reproducibility. Moreover, the contaminating molecule that nonspecifically binds to the QCM surface can conspicuously interfere with the QCM-based assay. In order to respond to the challenges, we designed a QCM-based sensor, in which an aptamer was combined to QCM surface to improve the selectivity and Au-NPs were used as an amplifier for the amplification of QCM signal to increase the sensitivity.

**Scheme 1.**
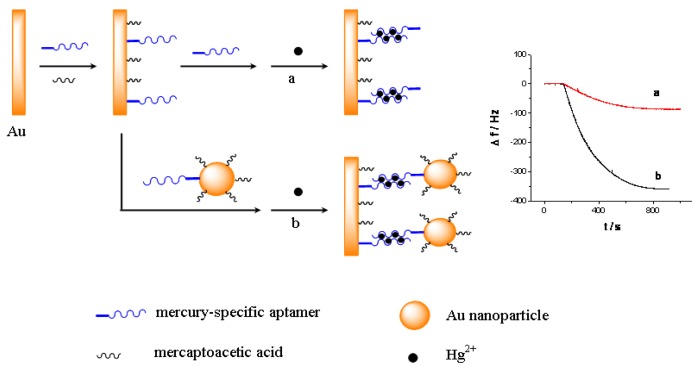
Schematic procedure of the sensing interface for detection of Hg^2+^ based on (**a**) aptamer and (**b**) aptamer-(Au-NPs) conjugates.

As shown in Scheme 1, a short thiolated MSA is immobilized on the QCM electrode surface as the capturing probe, and the MSA linked gold nanoparticles is used as the linking probe in solutions. Subsequently, in the presence of Hg^2+^, they can form sandwich structural T-Hg^2+^-T base pairs through the Hg^2+^ mediated coordination. Consequently, the use of Au-NPs can amplify the detection signal, which enhances the sensitivity for the QCM-based assay. Furthermore, because of the strong binding with Hg^2+^ [29], cysteine can disrupt the T-Hg^2+^-T sandwich structure, and therefore the designed QCM-based sensor can be regenerated in cysteine solution and be reused for multiple times. Particularly, by combining the QCM-based sensor with a flow injection system [30], the continuous and repeated assay of mercury can be realized easily.

## 2. Experimental

### 2.1. Reagents and Instruments

The mercury-specific aptamer was synthesized by Shanghai Kehua Bio-Engineering Co. Ltd. (Shanghai, China). The sequence was HS-5′-(CH_2_)_6_-TTTT-3′. 6-Mercapto-1-hexanol, HAuCl_4_, cysteine, hexaammineruthenium(III) chloride, potassium ferricyanide and tris(2-carboxyethyl)phosphine hydrochloride (TCEP) ware purchased from Aldrich (St. Louis, MO, USA) and used as received. Hg^2+^ stock solution (0.1 M) was prepared by dissolving HgCl_2_ with 0.5% HNO_3_ [12]. The Hg^2+^ stock solution was diluted to desired concentration with 0.05 M Tris-HCl buffer (pH 7.4) containing 0.1 M NaCl. All other reagents were of analytical grade and used without further purification. 0.05 M Tris-HCl buffer (pH 7.4) and 0.1 M phosphate buffered saline (PBS) (pH 7.4) were prepared according to standard procedures. All solutions were prepared with Milli-Q water (>18.2 MΩ·cm^−1^).

The QCM was performed on a CHI 440A quartz crystal microbalance electrochemical workstation (Shanghai Chenhua Ltd., Shanghai, China). A 7.995 MHz AT-cut quartz crystal (13.7 mm diameter) covered with gold electrodes (Φ 5 mm) was mounted levelly in a homemade Taflon holder. This Taflon holder was linked with a six-channel BT100K peristaltic pump.

The scanning electron microscopy (SEM) images used to characterize the morphology of the QCM electrode surface were obtained on an S-4800 microscope (Hitachi Ltd., Tokyo, Japan). Electrochemical impedance spectroscopy (EIS) and cyclic voltammetry (CV) were carried out with a CHI660c electrochemical workstation (Shanghai Chenhua Ltd., Shanghai, China). CV and EIS were performed in 5 mM [Fe(CN)_6_]^3−/4−^ solution which was purged with highly purified nitrogen at least 5 min. All the measurements were carried out at room temperature.

### 2.2. Direct Immobilization of the Mercury-Specific Aptamer Capturing Probe on QCM Electrode

The QCM gold electrodes were cleaned with phiranha solution [H_2_O_2_/H_2_SO_4_ 1:3 (v/v)] for 1 min, rinsed with copious amounts of purified water and ethanol, and dried with a gentle flow of nitrogen in order to remove grease and other pollutants from the electrode surface. Prior to the immobilization of the electrodes, aptamer stock solution (0.1 mM) was reduced in 10 mM TCEP for 2 h to cleave disulfide bonds. The resulting solution was then diluted with Tris-HCl buffer (pH 7.4) to the desired concentration (from 0.5 to 50 μM). Then, the QCM electrode was incubated in the aptamer solution for 16 h in the dark at 4 °C. Following incubation, the electrode was rinsed with water and then immersed in an aqueous solution of 1 mM 6-mercapto-1-hexanol for 1 h to passivate the electrode surface [31]. As a final step, the electrode was rinsed with water, dried with nitrogen, and stored at 4 °C prior to use.

### 2.3. Preparation of the Mercury-Specific Aptamer Linked with Au-NPs

Au-NPs were prepared according to a previously reported method with a slight modification [32,33]. In detail, solutions of HAuCl_4_ and trisodium citrate were filtered through a 0.8 μm microporous membrane filter prior to use. 1% Trisodium citrate (2.5 mL) was added to boiling 0.25 mM HAuCl_4_ solution (50 mL) and stirred for 10 min at the boiling point. The resulting solution was allowed to cool to room temperature. Concentration of the as-prepared Au-NPs was determined by UV-vis spectroscopy using Lambert-Beer's law (molar extinction coefficient of Au-NPs is 2.7 × 10^8^ M^−1^·cm^−1^ at λ_520_) [34]. The as-prepared Au-NPs were further co-modified with the MSA probe. Briefly, Au-NPs (5 mL, 50 nM) was incubated with a MSA probe (100 μL, 100 μM) for 16 h at room temperature. The final mixture was slowly brought up to a final salt concentration of 0.1 M NaCl, 10 mM TCEP, 1 mM 6-mercapto-1-hexanol and 10 mM PBS (pH 7.4) and allowed to age for 40 h. Centrifugation was performed at 14,000 rpm for 40 min in order to remove excessive MSA. The precipitate was washed with 0.1 M NaCl, 10 mM PBS buffer (pH 7.4) solution, recentrifuged, and finally dispersed in 10 mM Tris-HCl buffer (pH 7.4) containing 0.3 M NaCl for the further use. The as-prepared MSA-(Au-NPs) was denoted as the linking probe 2. In control experiment, the original MSA was denoted as linking probe 1.

## 3. Results and Discussion

### 3.1. Characterization of the Sensing Interface

**Figure 1.**
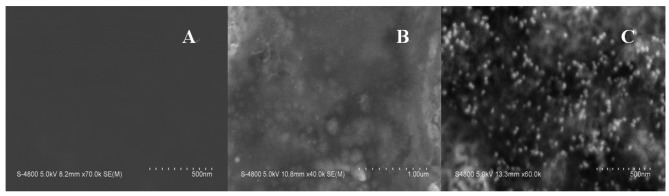
SEM images of (**A**) the bare Au electrode surface; (**B**) the Au electrode surface after hybridizing capturing probe with linking probe 1 in 0.5 μM Hg^2+^ solution and (**C**) the Au electrode surface after hybridizing capturing probe with linking probe 2 in 0.5 μM Hg^2+^ solution.

**Figure S1.**
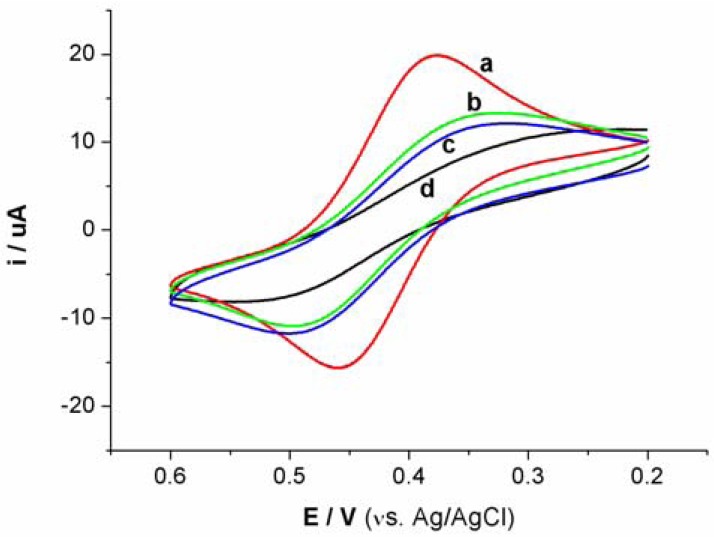
Characterization of the sensing interface by CV. (**a**) bare electrode; (**b**) after immobilization of capturing probe; (**c**) incubation with 1 μM linking probe 2 solution without Hg^2+^ for 30 min; (**d**) incubation with 1 μM linking probe 2 solution containing 0.5 μM for 10 min.

The principle of the sensor is based on the mass change on the sensing interface. In order to obtain the sensing signal, the designed aptamer must be first immobilized on the sensing interface as a capturing probe. Then, in the presence of Hg^2+^, a sandwich structure between the capturing probe and the linking probe is formed through the Hg^2+^ mediated T-Hg^2+^-T base pairs. SEM was used to characterize the sandwich structure on formed the sensing interface. Figure 1 shows SEM images of different interfaces after hybridizing in 0.5 μM Hg^2+^ solution. Figure 1(A) is a bare electrode surface. Figure 1(B) is the sensing interface after hybridization between the capturing probe and linking probe 1. In contrast, Figure 1(C) is the interface after hybridization between the capturing probe and linking probe 2. Comparing Figure 1(C) with 1(B), it is obvious that a large number of Au-NPs were successfully linked on the sensing interface through linking probe 2, which would result in a larger mass change on the QCM-based sensor in the same Hg^2+^ solution. Therefore, the frequency response (Δf) should remarkably increase compared with using linking probe 1. Consequently, the sensitivity of the QCM-based Hg^2+^ sensor can be significantly improved by using Au-NPs as the signal amplifier. In addition, electrochemical techniques including CV and EIS were utilized to characterize the steps of modified electrode and sensing for Hg^2+^. The results were shown in Figure S1–S2 in supplement information. The electrochemical data also demonstrate that the sensor was successfully prepared.

### 3.2. The Response of QCM-Based Sensor to Hg^2+^

**Figure 2.**
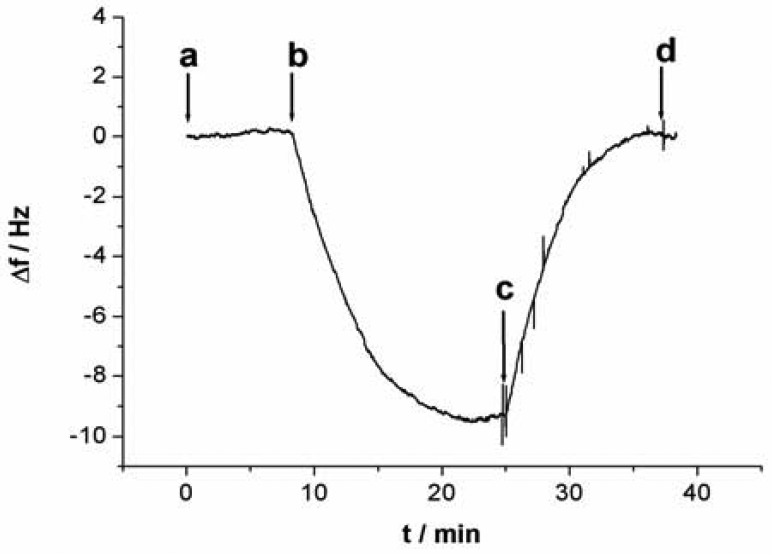
The response of QCM-based sensor to Hg^2+^. (a,b) section: 1 μM linking probe 2 solution was injected at a flow rate of 20 μL·min^−1^ for 600 s; (b,c) section: 5 nM Hg^2+^ solution was injected into the QCM cell; (c,d) section: 10 mM Tris-HCl buffer (pH 7.4) containing 50 mM cysteine was injected at a flow rate of 25 μL·min^−1^ for 900 s.

**Figure S3.**
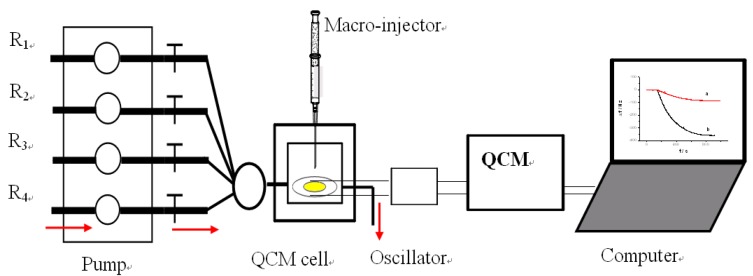
Schematic representation of the QCM-based sensor for detection of the Hg^2+^ (R_1_: Tris-HCl buffer; R_2_: linking probe 1; R_3_: linking probe 2; R_4_: cysteine).

All QCM experiments were performed in a QCM cell, in which the quartz crystal Au electrodes were modified with the capturing probe. The device for Hg^2+^ detection is presented in supplement information as Figure S3. The experimental process can be described as follows: 10 mM Tris-HCl buffer (pH 7.4) containing 0.1 M NaCl was injected firstly into the QCM cell with a flow rate of 20 μL/min and the crystal frequency was then measured as a background signal. After the signal change is less than 1 Hz of drift in 60 s, the experiment can be continued. 1 μM linking probe (1 or 2) solution was followed at a flow rate of 20 μL·min^−1^ and the signal (ΔF) of the QCM-based sensor was recorded. The response of the sensor to Hg^2+^ was shown in Figure 2. As can be seen in the a-b sections of Figure 2, this step was stopped after 600 s, Then, Hg^2+^ was injected into the QCM cell by using micro-injector and the solution in QCM cell was remained to quiet until a stable signal (ΔF) was obtained (typically, about 900 s). In this process, Hg^2+^ was captured with a sandwich structure, T-Hg^2+^-T, formed through the capturing probe, Hg^2+^ and linking probe (1 or 2). This step provided a readout signal and frequency change for quantitative detection of mercury ions, as shown in the b-c sections of Figure 2. Subsequently, 10 mM Tris-HCl buffer (pH 7.4) containing 50 mM cysteine was followed at a flow rate of 25 μL·min^−1^. In this step, the sandwich structure T-Hg^2+^-T was dissociated and Hg^2+^ was released from the sensing interface. Such that, the signal of the sensor recovered gradually to the level before Hg^2+^ was injected (c-d sections of Figure 2), indicating that the sensing interface of sensor has been renewed. The regenerated sensor could be reused again and the above process could be repeated continuously. This strategy provides a facility for the continuous determination of Hg^2+^.

### 3.3. Amplification Effect of Au-NPs

As a highly sensitive mass sensor, the QCM signal depends on the mass change on its sensing interface. For the same amount of target, if the mass change on the sensing interface can be increased greatly, the sensitivity of detection should be improved remarkably. In this work, Hg^2+^ was detected through a specific sandwich structure formed by the capturing probe, Hg^2+^ and the linking probe, which caused the mass change on the sensing interface [35]. Au-NP was employed to link to the linking probe to get much more mass change on the sensing interface.

**Figure 3.**
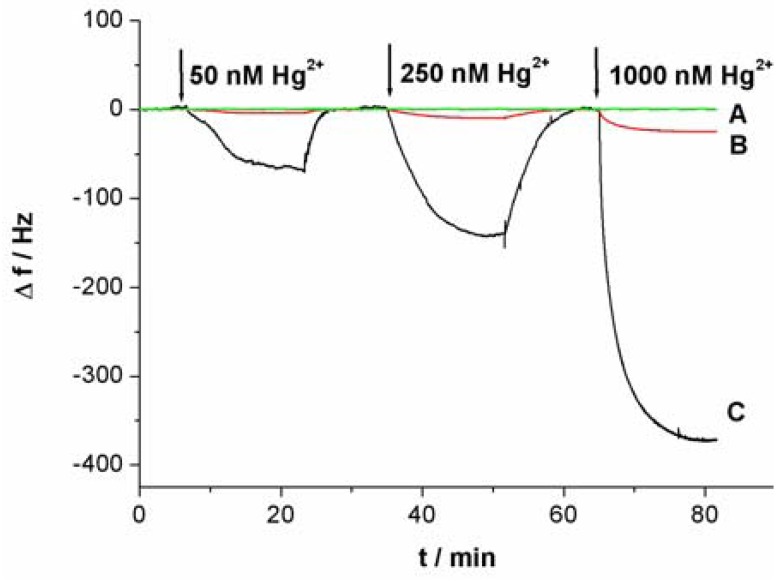
Signal responses of the QCM-based sensor to Hg^2+^ in different linking probe solution. (**A**) Tris-HCl buffer without linking probe; (**B**) linking probe 1; (**C**) linking probe 2.

Figure 3 shows the response curves of the QCM-based sensor to different concentrations of Hg^2+^. As shown in Figure 3(A), without linking probe, no frequency change (Δf) can be observed. In Figure 3(B), an obvious Δf can be detected due to the use of linking probe 1. When linking probe 2 was used instead of linking probe 1, the response signal, Δf, increases greatly, about 15.2-fold under the same Hg^2+^ concentration, as shown in Figure 3(C). Since a large mass of Au-NP was immobilized on the linking probe, an amount of Au-NPs were loaded on the sensing interface through the formation of the sandwich structure, which led to more mass change on the sensing interface, as shown in Figure 1(C). Thus, the detected signal was greatly amplified by the Au-NPs.

### 3.4. Effects of Capturing Probe Density on Sensor Performance

**Figure 4.**
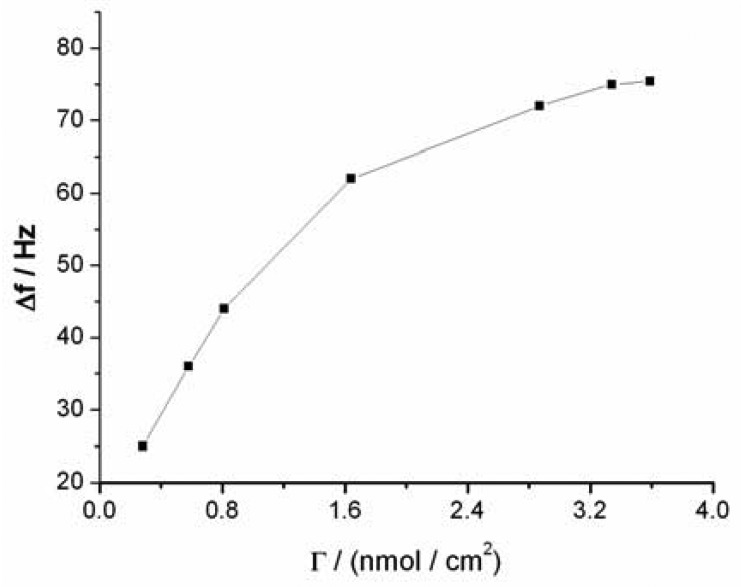
The dependence of the sensor signals on the surface density in the presence of 50 nM Hg^2+^ solution.

The sensitivity of the QCM-based sensor is dependent on the amount of sandwich structures formed under the same Hg^2+^ concentration, which depends in turn on the surface density of capturing probe molecules on the sensor surface. The dependence of the sensor signal on the surface density of capturing probe in same Hg^2+^ solution is depicted in Figure 4, which shows that a low sensor sensitivity was obtained when a low density of capturing probe was used. The sensitivity of the sensor increases with the increase of density of the capturing probe. When the density reached about 3.25 nmol·cm^−2^, the response signal reaches the maximum, which is ascribed to the saturation of immobilized capturing probe on the sensor surface (the surface density was calculated on the basis of the Δf measured during QCM experiments) [36,37]. The surface density of capturing probe molecules on the sensor surface was also calculated to be 3.75 × 10^14^ molecules·cm^−2^ by Tarlov's electrochemical method [31]. This value is less than 3.25 nmol·cm^−2^ (1.96 × 10^15^ molecules·cm^−2^) by the QCM method. The surface density of probe calculated by QCM may also contain contributions from the thimbleful of adsorbed water on the sensor surface. Compared with other reports employing hairpin aptamer ((6.57 ± 0.55) × 10^13^ molecules·cm^−2^) [38], the surface density of our sensor is larger, which is likely attributable to the use of short apatmers (only containing four thymines). The high surface density should greatly improve the sensitivity of the sensor and significantly reduce nonspecific adsorption and contaminant trapping.

### 3.5. Sensitivity, Specificity and Reusability of the QCM-Based Sensor

**Figure 5.**
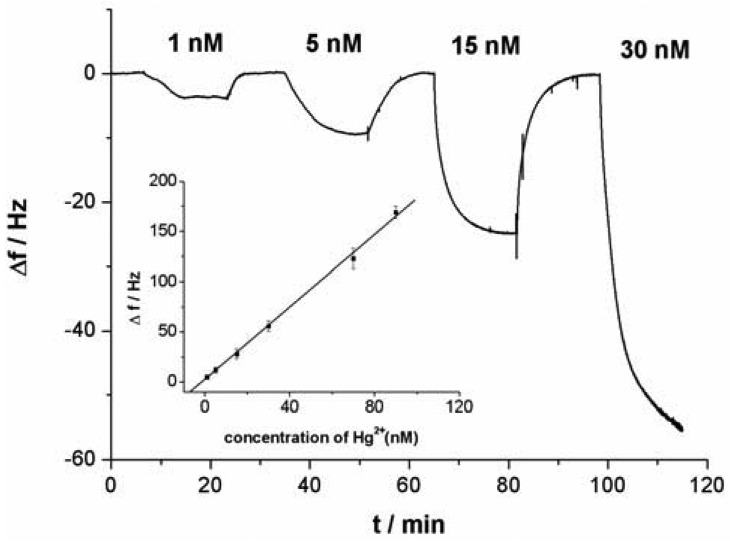
The dependence of the response signal of QCM-based sensor on Hg^2+^ concentration. Inset: the linear relationship between the Δf and the Hg^2+^ concentrations.

As shown in Figure 3(C), with the increase of Hg^2+^ concentration, the response signal of the sensor (Δf) obviously increases. As a result, the relationship between the response signal and the Hg^2+^ concentration can be established. Under the optimal conditions in which linking probe 2 was used, a linear relationship between the response signal Δf and Hg^2+^ concentrations was obtained (shown in an inset of Figure 5). The Δf increased linearly with Hg^2+^ concentrations in the tested range from 0.5 nM to 100 nM. According to the experimental data, a linear equation could be calculated: Δf = 1.82C + 1.60 (C is the concentration of the target Hg^2+^, 10^−9^ M; Δf is the signal of sensor, Hz) with a relationship coefficient of 0.9986. The detection limit was evaluated to be 0.24 ± 0.06 nM. Under the same conditions expect for the using of Au-NPs as amplifier, in which linking probe 1 was used, the detection limit was about 0.3 μM. The detection limit for Hg^2+^ detection was increased remarkably, about three orders of magnitude, by using Au-NPs as amplifier. Compared with data reported in the literature for other aptamer-based Hg^2+^ sensors, the detection limit in this work was lower. Table 1 summarizes the sensitivity reported for several aptamer-based Hg^2+^ sensors. The present Au-NPs amplification strategy reveals a superior sensitivity as compared to protocols of aptamer-based Hg^2+^ sensors. It is clear that the detection limit is dependent on the sensitivity of assay and the stability of detecting signal. The lower detection limit suggests that both sensitivity and stability of the sensor have improved when Au-NP was used as the amplifier.

**Figure 6.**
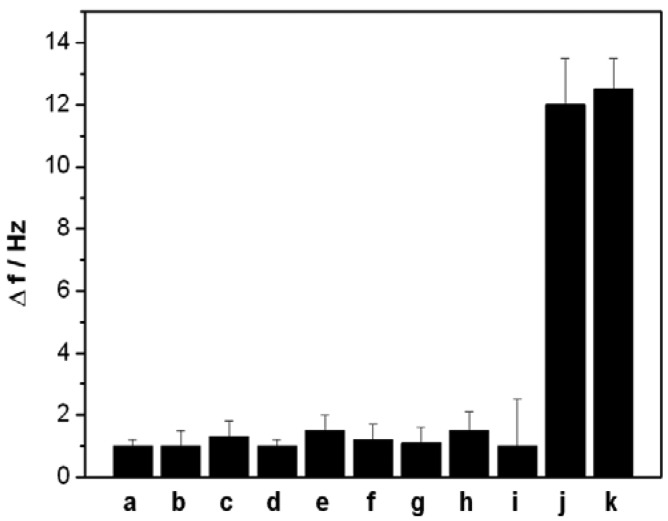
Response of the QCM-based sensor to different ions: (**a**) 0.5 mM Ca^2+^; (**b**) 0.5 mM Mg^2+^; (**c**–**h**) Cu^2+^, Cd^2+^, Pb^2+^, Zn^2+^, Ni^2+^, Ag^+^(0.5 μM, each); (**i**) Au^3+^ (0.1 μM) (**j**) 5 nM Hg^2+^; (**k**) the mixture of Ca^2+^, Mg^2+^ (0.5 mM, each), Cu^2+^, Cd^2+^, Pb^2+^, Zn^2+^, Ni^2+^, Ag^+^(0.5 μM, each); and 5 nM Hg^2+^.

In the present work, a short ss-DNA containing only four thymine (T) bases was designed as an aptamer to selectively capture Hg^2+^, which can selectively and strongly bind Hg^2+^ and form a stable ds-DNA through metal-mediated T-Hg^2+^-T structure. To investigate the specificity of the designed QCM-based sensor, we also challenged this sensor with nine interference ions: Ca^2+^ and Mg^2+^ (0.5 mM, each), Cu^2+^, Cd^2+^, Pb^2+^, Zn^2+^, Ni^2+^ and Ag^+^(0.5 μM, each), Au^3+^(0.1 μM). Figure 6 depicts the signal response of the sensor to the above mentioned ions. Compared with the significant frequency change (12 Hz) of 5 nM Hg^2+^, the 100-fold or higher concentrations other interference ions, except for Au^3+^, only produced a slight frequency change (<2 Hz), suggesting that the present sensor has highly selectivity owing to the specific coordination between T bases and Hg^2+^ ions. However, the interference of Au^3+^ was noted to be relatively great than that of other common metal ions.

**Figure 7.**
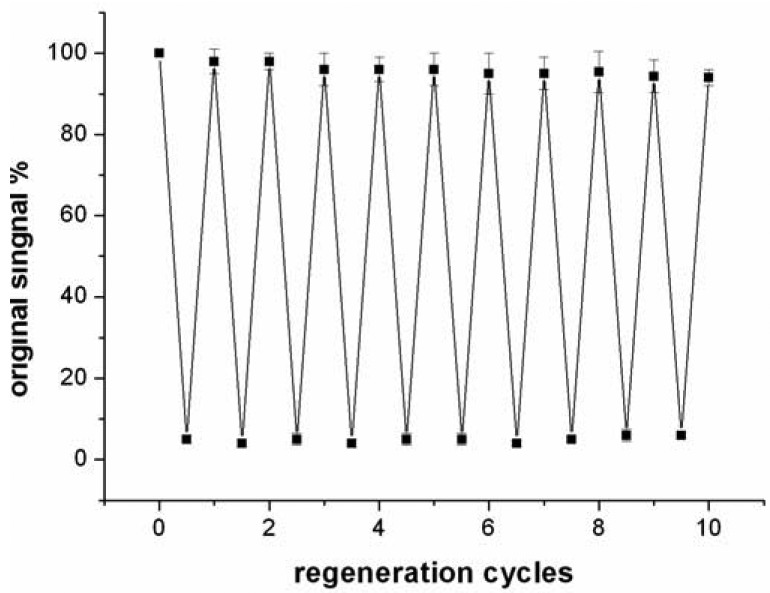
Reusability of the QCM-based Hg^2+^ sensor challenged with 5 nM Hg^2+^ and washed with 50 mM cysteine.

Compared to antibody-based affinity biosensors, aptamer-based sensors provide an advantage of chemical stability [11]. The chemical stability of aptamer ensures that the sensor can be regenerated under proper conditions. In this work, the sensor was regenerated with 50 mM cysteine solution, because the sandwich structure of T-Hg^2+^-T can be disrupted by cysteine. The sensor was firstly challenged with 5 nM Hg^2+^ to obtain a response signal. Then, the sensor was washed with a flowing cysteine solution and the signal of blank solution was determined again. The above procedure was repeated continuously for 10 times and the results were presented in Figure 7.

The data suggested that the response signal to the same concentration Hg^2+^ only attenuates about 5.7% after 10 cycles and the blank signal almost remains unchanged, implying that the sensor can be regenerated and reused at least 10 times. In addition, when the sensor was stored in pH 7.4 Tris-HCl buffer at 4 °C, 97% and 91% response signal are retained after 7 days and 15 days, respectively. Linking probe 2 is also stable within 15 days when it is stored in pH 7.4 Tris-HCl buffer at −10 °C.

### 3.6. Detection of Hg^2+^ in Real Samples

To validate the application of our sensor in real water samples, it was used to determine the levels of Hg^2+^ in waste and tap water samples. Prior to water sample analysis, the waste water samples were filtered through a 0.45 μm membrane. As described in the earlier section, 50 μL of waste water or tap water spiked with different concentration of Hg^2+^ was injected into the QCM cell. The concentration of Hg^2+^ was determined by applying the standard curve method and the results were summarized in Table 2. It can be seen that mean recoveries range between 96.8% and 101.6% with relative standard deviations under 4.2%. The results indicate that the QCM-based sensor was highly accurate, precise, and reproducible, and can be used for the direct analysis of traces of mercury in real samples.

## 4. Conclusions

The main advantage of the proposed QCM-based sensor is that it is label-free, which provides great simplicity for the construction of a biosensor, although the sensitivity and stability of the detected signal need further improvement. In this work, a sensitive QCM-based sensor for selective detection of Hg^2+^ was constructed by using Au-NPs as the signal amplifier. Compared with sensors prepared without a signal amplifier, our sensor displayed about three orders of magnitude increase in the detection limit. By combining with flow injection and the regeneration, Hg^2+^ can be continuously determined with a low detection limit of 0.24 nM. This strategy could also be used for constructing other QCM-based sensors for various targets. Due to its label-free nature, the constructed sensor should be applied conveniently in different fields.

## Figures and Tables

**Table 1.** Comparison of the sensitivity of different Hg^2+^ sensors.

**Aptamer-based Hg^2+^ sensor**	**Limit of detection (nM)**	**Reference**
fluorometric assay based on the allosteric DNAzyme catalytic beacons	2.4	[39]
fluorometric assay based on aptamer/reporter conjugates	10	[13]
fluorometric assay based on the aptamer beacon	40	[16]
fluorometric assay based on AuNPs quenched fluorophore modified aptamer	40	[14]
colorimetric assay based on the aggregation of thiolated aptamer modified Au NPs	100	[40]
colorimetric assay based on AuNPs probes and thrombin-binding aptamer	200	[14]
colorimetric assay based on the salt-induced aggregation of nonmodified aptamer stabilized AuNPs	10	[41]
square wave voltammetry on AuNPs amplified aptamer based sensor	0.5	[12]
QCM aptamersensor based on signal amplification with gold nanoparticles	0.24	Present study

**Table 2.** Results obtained in analysis of waste and tap water samples with the QCM-based sensor.

**Concentration (μg·L^−1^)**									

**Sample**	**Added**	**Found**					**Avg. found**	**RSD, %**	**Recovery, %**
Waste water	0	5.8	6.2	6.3	6.4	5.9	6.10	4.2	-
	5	10.7	11.4	11.6	10.6	11.1	10.08	3.8	101.6
	10	15.1	16.2	15.3	16.6	16.2	15.78	3.3	96.8
Tap water	0	Not found					-	-	-
	10	9.6	10.4	9.7	9.9	10.1	9.94	3.2	99.4
	20	21.0	20.1	19.8	19.6	19.8	20.06	2.7	100.3

**Figure S2.**
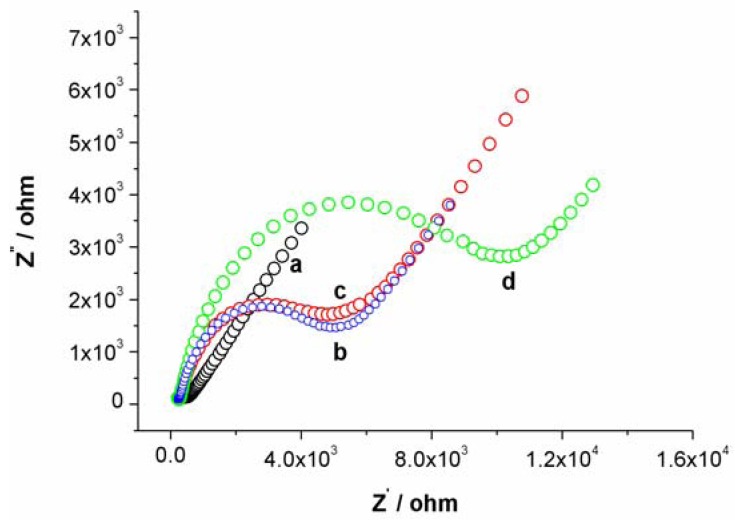
Characterization of the sensing interface by EIS. The procedure is the same as Figure S1.

## Appendix

### CV and EIS Characterizations of Sensing Interface

Cyclic voltammetry (CV) and electrochemical impedance spectroscopy (EIS) were utilized to characterize the steps of modified electrode and sensing for Hg^2+^. At bare Au electrode, a couple of well redox peaks of [Fe(CN)_6_]^4−/3−^ can be observed, as shown in Figure S1(a). Then, the Au electrode was incubated in an aptamer solution for 16 h. Following incubation, the electrode was washed with water and immersed in an aqueous solution of 1mM mercaptoacetic acid for 1 h to remove nonspecifically adsorbed aptamer molecules and to passivate the electrode surface. Subsequently, the Au electrode immobilized with MSA and MCA was used as a work electrode and CV experiment was performed in the same [Fe(CN)_6_]^4−/3−^ solution. As shown in Figure S1(b), the peak currents obviously decreases and the difference between cathodic and anodic peak potential increases. The negative charge of MSA and mercaptoacetic acid on electrode surface prohibited the electron transfer of [Fe(CN)_6_]^4−/3−^ at electrode interface. Following this step, the electrode was immersed in 1 μM linking probe 2 solution (containing 1 μM linking probe 2, 10 mM Tris-HCl, and 0.1 M NaCl) for 30 min. The obtained CV response, Figure S1(c), is very similar to S1b, revealing that the electrode surface does not obviously change. However, when the electrode was immersed into linking probe 2 solution containing Hg^2+^ for 10 min, hardly any redox peaks of [Fe(CN)_6_]^4−/3−^ can be observed, as shown in Figure S1(d). This likely is attributed to the formation of the sandwich structure of T-Hg^2+^-T, which further block the electron transfer of [Fe(CN)_6_]^4−/3−^ at electrode surface. These results also can be demonstrated by EIS, as shown in Figure S2.
